# The Relationship between Ischemic Stroke Patients with and without Retroflex Tongue: A Retrospective Study

**DOI:** 10.1155/2017/3195749

**Published:** 2017-03-06

**Authors:** Yung-Sheng Huang, Mu-Chien Sun, Po-Chi Hsu, Yu-Liang Chen, John Y. Chiang, Lun-Chien Lo

**Affiliations:** ^1^Department of Traditional Chinese Medicine, Changhua Christian Hospital, Changhua, Taiwan; ^2^Stroke Center and Department of Neurology, Changhua Christian Hospital, Changhua, Taiwan; ^3^Graduate Institute of Chinese Medicine, College of Chinese Medicine, China Medical University, Taichung, Taiwan; ^4^Department of Healthcare Administration and Medical Informatics, Kaohsiung Medical University, Kaohsiung 80708, Taiwan; ^5^Department of Computer Science and Engineering, National Sun Yat-sen University, Kaohsiung 80424, Taiwan; ^6^Graduate Institute of Statistics and Information Science, National Changhua University of Education, Changhua, Taiwan

## Abstract

*Background*. Patients suffering from stroke exhibit different levels of capability in retroflex tongues, in our clinical observation. This study aims to derive the association of tongue retroflexibility with the degree of severity for stroke patients.* Methods*. All ischemic stroke patients were collected from August 2010 to July 2013 in the Stroke Center, Changhua Christian Hospital, Taiwan. All participants underwent medical history collection and clinical examination, including tongue images captured by ATDS. Statistical analysis was performed to compare the differences of ischemic stroke patients with and without retroflex tongue.* Result*. Among the total of 308 cases collected, 123 patients cannot retroflex their tongues, that is, the non-RT group. The length of stay in the non-RT group, 32.0 ± 21.5, was longer than those of the RT counterparts, 25.9 ± 14.4 (*p* value: 0.007). The NIHSS on admission, 14.1 ± 7.8 versus 8.9 ± 5.2, was higher and the Barthel Index upon admission, 18.6 ± 20.7 and 35.0 ± 24.2, was lower for the non-RT patients than that of the RT counterparts. Also, the non-RT patients account for 60.2% and 75.6% for Barthel Index ≤ 17 and NIHSS ≥ 9, respectively.* Conclusion*. The stroke patients in non-RT group showed significantly poor prognosis and were more serious in the degree of severity and level of autonomy than RT group, indicating that the ability to maneuver tongue retroflex can serve as a simple, reliable, and noninvasive means for the prognosis of ischemic stroke patients.

## 1. Introduction

Stroke is a disease leading to high mortality and morbidity around the world. In western society, an average of 0.2% population had stroke [1]. In fact, stroke, with high recurrence rate, is a very common death-causing disease, second only to ischemic heart disease, and keep increasing annually [2]. In the US, there were approximately 6.6 million stroke patients with age above 20 in 2013 alone, and this figure will grow up to an amount of 3.4 million patients by 2030 with an increase of 20.5% [3]. In a retrospective study of 10,399 stroke patients in 2002, the recurrence rate for one month, six months, one year, and four years is 1.8%, 5%, 8%, and 18.1%, respectively [4]. A great impact in personal life quality, work and finance is associated with the occurrence of stroke accident. Stroke can be divided into two main types, namely, ischemic and hemorrhagic strokes, with the ischemic stroke, the intracranial hemorrhage, and subarachnoid hemorrhage accounting for 87%, 10%, and 3%, respectively [5].

Other than thrombolytic therapy and craniotomy, the treatment focuses on rehabilitation of stroke sequela. Acupuncture intervention has demonstrated obvious benefits to the stroke patients [6, 7]. A systemic research shows that MLC601 (NeuroAiD®), composed of Chinese herbs, can improve the life independent capability after stroke [8]. Another systemic research shows that acupuncture has improvements in neurodamage and function disability after stroke, such as dysphagia [9], indicating that TCM intervention can be a good approach in treating stroke.

According to TCM theory, tongue diagnosis is one of the main diagnostic methods. Researches about tongue has becoming more popular. For example, The appearance of tongue coating has shown to be closely linked to the pathological changes of the digestive system [10, 11]. Tongue deviation is one of the important factor in predicting stroke, and reports shows its importance and specificity [12–14]. Using tongue diagnosis to standardize stroke patients can be a diagnostic criteria in the future [15]. All these indicate the simple, noninvasive, and intuitive tongue diagnosis deserves further exploration in the treatment of disease.

According to our clinical experience, stroke patients often encounter difficulty in curling up their tongues to allow a TCM practitioner inspecting the sublingual veins. Based on the capability in curling up tongue, a subject is assigned to either the retroflex tongue (RT) or the non-RT group. This study aims at linking the degree of severity of ischemic stroke with the ability of tongue retroflexibility. Subsequent prognosis and rehabilitation program can be devised through a simple and noninvasive tongue inspection to assess the degree of severity of a stroke patient.

## 2. Materials and Methods

This is a retrospective study, approved by Institutional Review Board of Changhua Christian Hospital (CCH), Taiwan (IRB number 150110). The stroke patients admitted to CCH and consulted TCM treatment from August 1, 2010, to July 31, 2013, were recruited. The tongue database was jointly collected by the Department of TCM and Stroke Center in CCH. The name, ID and medical record number of the subject were scrambled to protect individual privacy. The research team followed the guideline of IRB strictly during the period of this study.

### 2.1. Inclusion Criteria

Participants meeting the following criteria would be included:

1. Participants diagnosed as ischemic stroke (ICD-9: 433~438) by neurologist and approved by head CT or MRI examination
2. The period from ischemic stroke diagnosis to tongue examination less than 6 months
3. Age above 20 years old
4. Complete tongue diagnosis data
5. Agreement in joining this study

### 2.2. Exclusion Criteria

Participants meeting one or more of the following criteria would be excluded:

1. Hemorrhagic stroke (ICD-9: 430~432)
2. With history of ischemic heart disease, including acute myocardial infarction (ICD-9: 410) and other ischemic heart diseases (ICD-9: 411–414)
3. Unstable vital sign or unconsciousness
4. Unable to open mouth or risk of jaw dislocation
5. Unable to protrude tongue or with insufficient length protruded to allow capturing of tongue image
6. Cognitive impairment or unable to communicate

## 3. Autonomic Tongue Diagnostic System (ATDS)

**Figure 1:**
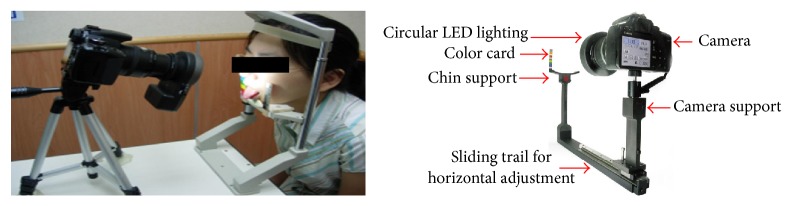
Image acquisition through the Automatic Tongue Diagnosis System, ATDS.

**Figure 2:**
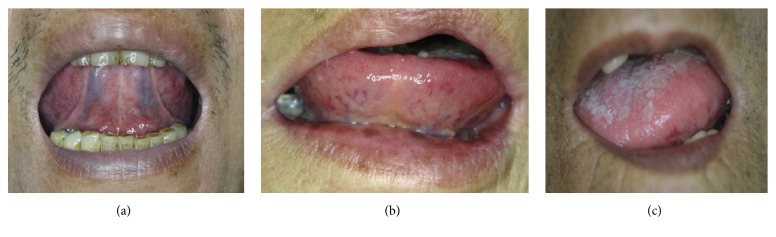
(a) Retroflex tongue; that is, a tongue can freely curl up and expose the sublingual veins for observation, (b) nonretroflex tongue with very limited tongue movability: no sublingual vein was observed, and (c) tongue slanted to one side, no curling up of tongue at all.

The tongue examination images were acquired and screened by professional tongue diagnosis technician. With the assistance of ATDS [16, 17], the color of tongue images captured was balanced to reduce the lighting interference from image-taking environment and inherent human visual error, as shown in Figure 1. Five experienced TCM practitioners, each with more than 5 years clinical experience, classified every tongue image into either retroflex tongue, i.e., the tip of tongue curled up to the upper lip while protruding, or nonretroflex tongue based on consensus reached. As illustrated in Figures 2(a)–2(c), a retroflex tongue represents a tongue can curl up freely and allow clear observation of the sublingual vein. On the other hand, the nonretroflex tongue corresponds to a patient who cannot curl tongue up to expose sublingual veins for visual inspection. The group of nonretroflex tongue includes both a tongue failing to curl up at all, and one moves inadequately to allow visual inspection of sublingual veins. The immobility often causes tongue slanting to one side with insufficient protruding length.

## 4. Data Collection

Data collected included age, sex, height, weight, ICD-9 code, the time period from ischemic stroke diagnosis to tongue examination, the admission date and discharge date, NIHSS score and Barthel Index score on admission, TCM tongue features, stroke-related diseases (diabetes, hypertension, hyperlipidemia, etc.), and laboratory data (HbA1c, T-cholesterol, triglyceride, HDL, etc.).

## 5. Statistical Analysis

All the analyses were conducted using SPSS 22.0 statistical software. Chi-square test or Fischer's exact test was utilized to analyze the nominal variables, while Independent-Samples *t*-test was employed for the continuous ones. Furthermore, the receiver operating characteristic (ROC) curve was used to investigate the relationship between tongue retroflexibility and NIHSS and Barthel Index. *p* value < 0.05 was considered statistically significant.

## 6. Results

A total of 308 stroke patients admitted to CCH and consulted TCM treatment from August 1, 2010, to July 31, 2013, were recruited. The retroflexibility of tongue served as the discriminating criterion to segment the stroke cases into two clusters, that is, RT and non-RT groups. Age, height, weight, BMI, gender, history of stroke-related diseases (diabetes, hypertension, hyperlipidemia, and etc.), time of first incidence of stroke, tongue deviation and laboratory data (HbA1c, T-cholesterol, Triglyceride, HDL, and etc.) bear no significant difference between these two groups, as listed in Table 1.

The ischemic stroke patients who cannot retroflex their tongues had longer length of stay than the RT counterparts, 32.0 ± 21.5 days versus 25.9 ± 14.4 days (*p* value: 0.007). The NIHSS score on admission was higher in non-RT group than that of RT one, 14.1 ± 7.8 versus 8.9 ± 5.2 (*p* value < 0.001). The Barthel Index on admission was lower in non-RT group also, with 18.6 ± 20.7 versus 35.0 ± 24.2, as shown in Table 2.

**Figure 3:**
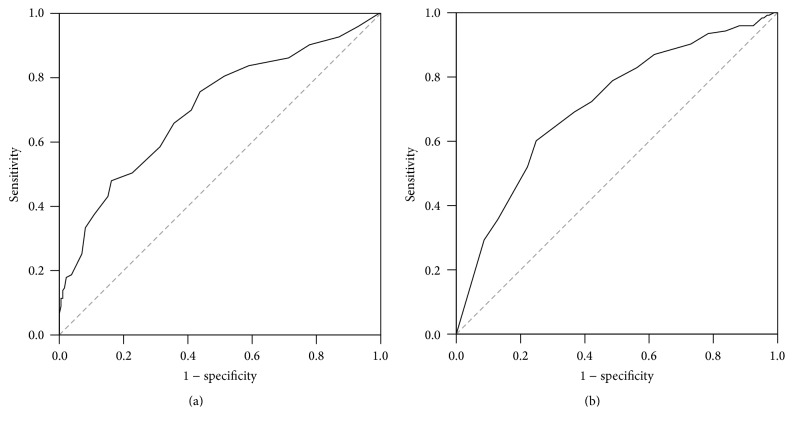
The receiver operating characteristic curve (ROC curve) between retroflex tongue and (a) NIHSS score values and (b) Barthel Index.

We applied receiver operating characteristic curve (ROC curve) to assess the relationship between retroflex tongue and NIHSS score, Barthel Index of ischemic stroke patients. Youden's index (sensitivity + specificity − 1) showed highest values 0.318 and 0.353 and NIHSS score > 9 and Barthel Index < 17, respectively. The sensitivity and specificity were 75.6% and 56.2% under the diagnostic criteria of NIHSS ≥ 9. The area under curve was 0.703. The sensitivity and specificity were 60.2% and 75.1% under the diagnostic criteria of Barthel Index ≤ 17, and the area under curve was 0.712. The data was presented in Table 3, and the ROC curves were presented in Figure 3.

The two variables, NIHSS score > 9 and Barthel Index < 17, were applied to conduct statistical comparison, and the results showed significant statistical differences (*p* value < 0.001). The ratios of non-RT and RT groups were 75.6% and 43.8% in NIHSS ≥ 9 and 60.2% and 24.9% in Barthel Index ≤ 17, respectively. The data was presented in Table 4.

## 7. Discussion

The importance of TCM of four examinations in disease diagnosis and prognosis prediction has been gaining more recognition. A retrospective study pointed out the following eight items, namely, TCM symptoms within three days, NIHSS scale, age, diabetes mellitus history, anxiety, irritability, and tinnitus, reach the cutoff point of 9.5, a poor outcome can be predicted in the 90-day prognosis of ischemic stroke patients [18]. Another study showed that tongue biting or not can effectively distinguish epileptic seizures from syncope (OR: 12.26; 95% CI: 3.99–37.69) [19]. These aforementioned researches indicate that tongue appearance reflects the abnormality of the body. Tongue diagnosis can serve as an effective and noninvasive means of examination to decrease the examination time and cost, increase the ability of diagnosis and the reliability of prognosis. For example, There was a pioneering study regarding the relationship between ischemic stroke and sublingual vein at the back of tongue. The NIHSS (5.28 ± 4.38 versus 10.57 ± 9.58, *p* < 0.001) was lower, while Barthel Index score (67.61 ± 29.29 versus 54.64 ± 36.23, *p* = 0.015) was higher in ischemic stroke patients with abnormal sublingual veins than with the normal ones [20]. It demonstrates that tongue diagnosis is taken more seriously.

According to our clinical observation, patients suffering from stroke exhibit different levels of capability in retroflex tongues. To our knowledge, this is the first study to evaluate the relationship between retroflex tongue and stroke.

The NIHSS score for non-RT group was higher than that of RT one (14.1 ± 7.8 and 8.9 ± 5.2), corresponding to a higher degree of severity of ischemic stroke in patients within the non-RT group. The Barthel Index, often served as an assessment of patient's dependency of life, was lower than that of RT counterpart, representing a higher degree of life dependency in ischemic stroke patients who cannot curl up their tongues. And the mean of Barthel Index between non-RT and RT group can be represented as total and severe dependency (18.6 ± 20.7 and 35.0 ± 24.2) [21].

The Receiver Operating Characteristic curve (ROC curve) was applied to derive the best cutoff point in NIHSS and Barthel Index. According to Youden's index, the best cutoff points are NIHSS ≥ 9, and Barthel Index ≤ 17. According to the sensitivity and specificity of ROC, Barthel Index could serve as a better alternative than NIHSS. If NIHSS = 9 and Barthel Index = 17 are used as cutoff points, patients in non-RT group account for 75.6% by NIHSS ≥ 9. These ratio are higher than RT group (43.8%). The point Barthel Index ≤ 17 has higher difference, and the ratios in non-RT and RT groups are 60.2% and 24.9%, respectively. That indicates 60% of non-RT group has Barthel Index ≤ 17, and 75.1% of RT group has Barthel Index > 17. By reviewing literatures, we found there was no standard definition in severity of NIHSS score. The severity are mostly defined as mild in <5 or 6, severe in >14 or 15 [22, 23], or ≥10 as severe stroke [24]. The latest definition of National Institutes of Health Stroke Scale (NIHSS) severity is mild NIHSS (≤8), moderate NIHSS (9–15), and severe stroke NIHSS (≥16) [25]. According to the literature, we could easily evaluated more than 60% of non-RT stroke patients are total dependency (Barthel Index defined as “total” dependency in ≤20) [21], and more than 70% of non-RT stroke patients are moderate to severe stroke. RT can be an indicator in evaluating the severity of stroke.

The anatomy of the corticohypoglossal projections and the clinical effect of lesions on them are not clear-cut [14]. The hypoglossal nerve (CN XII) controls the motor of tongue. The origin of hypoglossal nerve is hypoglossal nucleus, composed by motor fiber. The nerve extends from the anterior-lateral part of medulla to hypoglossal nerve, controlling eight muscles of tongue movement. These muscles are four extrinsic and intrinsic muscles. Except palatoglossus muscle is innervated by vagus nerve, the other seven muscles are innervated by hypoglossal nerve. The two main muscles responsible for the curling of tongue body are superior longitudinal muscle and styloglossus muscle [26]. Besides, genioglossus muscle which protrudes the tongue is mainly used in testing hypoglossal nerve. Once the nerve is damaged, the tongue would deviate to the side affected. Areas more liable to the damage caused by stroke included supranuclear, nuclear, or infranuclear locations [12]. Medullary lesions typically cause “crossed hemiplegia” with tongue weakness and deviation away from the hemiplegic side [27]. According to the anatomy, the retroflex tongue and tongue deviation should be induced by the damage of corticohypoglossal pathway. However, there is no statistical difference in tongue deviation between the RT and non-RT groups in our study, indicating that the damage of hypoglossal nerve in the traditional evaluation shows no statistical difference. It is worth exploring if there are other joint nerves controlling the tongue or other unknown factors, such as the location and severity of infarction cerebral cortex.

However, there are some limitations in this research. Whether stroke patients could perform retroflex tongue before stroke happening or not cannot be confirmed. As this research is a retrospective study, we cannot exclude these patients by interpret former tongue diagnostic images. Although there were no differences among basic data and ratio among retroflex tongue in two groups, we hope that retroflex tongue can be a simple and effective method in evaluating the severity of stroke.

## 8. Conclusion

The stroke patients in non-RT group showed significantly poor prognosis with longer length of stay and was more serious in the degree of severity and level of autonomy revealed through NIHSS and Barthel Index than RT group, indicating that the ability to maneuver tongue retroflex can serve as a simple, reliable, and noninvasive means for the prognosis of ischemic stroke patients.

## Figures and Tables

**Table 1:** The basic data of ischemic stroke patients between retroflex tongue and nonretroflex tongue groups.

Variable	Retroflex tongue		*p* value
	With (*N* = 185)	Without (*N* = 123)	
Gender, *N* (%)			0.121
Male, *N* (%)	115 (62.2)	87 (70.7)	
Female, *N* (%)	70 (37.8)	46 (29.3)	
Comorbidity			
DM, *N* (%)	76 (41.1)	50 (40.7)	0.940
HTN, *N* (%)	148 (80.0)	103 (83.7)	0.408
Hyperlipidemia, *N* (%)	100 (54.1)	60 (48.8)	0.364
First time stroke, *N* (%)	149 (80.5)	93 (75.6)	0.393
Tongue deviation, *N* (%)	35 (18.9)	24 (19.5)	0.897
Age, mean (SD)	68.0 (12.8)	70.4 (10.5)	0.085
Weight, mean (SD)	63.7 (13.5)	64.1 (12.1)	0.792
Height, mean (SD)	1.6 (0.1)	1.6 (0.1)	0.697
BMI, mean (SD)	24.3 (4.1)	24.4 (4.0)	0.825
HbA1C (%), mean (SD)	7.0 (2.1)	6.8 (2.0)	0.529
Total cholesterol (mg/dL), mean (SD)	184.8 (42.9)	174.3 (44.5)	0.071
Triglyceride (mg/dL), mean (SD)	123.3 (78.2)	121.3 (97.4)	0.859
HDL (mg/dL), mean (SD)	39.6 (17.4)	44.7 (26.1)	0.465

Student's *t-*test and Chi-square test or Fisher's exact test.

**Table 2:** The severity assessment of ischemic stroke patients between retroflex tongue and nonretroflex tongue groups.

Variable	Retroflex tongue		*p* value
	With (*N* = 185)	Without (*N* = 123)	
Length of stay (day), mean (SD)	25.9 (14.4)	32.0 (21.5)	0.007^*∗*^
NIHSS score^¡^, mean (SD)	8.9 (5.2)	14.1 (7.8)	<0.001^*∗*^
Barthel Index^¶^, mean (SD)	35.0 (24.2)	18.6 (20.7)	<0.001^*∗*^

Student's *t-*test and Chi-square test or Fisher's exact test; ^*∗*^*p* < 0.05 has significant statistical differences.

^¡^National Institutes of Health Stroke Scale (NIHSS) ranges from 0 to 42 scores; the higher the score means higher neurodamage. The NIHSS has a total of 15 items.

^¶^Barthel Index is a common life function scale, ranging from 0 to 100 scores. The lower the score, the higher the life-dependency. Barthel Index has five degrees; scores of 0–20 indicate “total” dependency, 21–60 “severe” dependency, 61–90 “moderate” dependency, 91–99 “slight” dependency, and 100 “total” independency. The cases collected mostly belong to severe dependency.

**Table 3:** The ROC curve of retroflex tongue and NIHSS and Barthel Index of ischemic stroke patients.

Criterion values and coordinates of ROC curve					Area under the ROC curve			
Value	Sensitivity	Specificity	+LR	−LR	Area	SE	95% CI	*p* value
NIHSS ≥ 9	0.756	0.562	1.727	0.434	0.703	0.031	0.643–0.764	<0.001^*∗*^
Barthel Index ≤ 17	0.602	0.751	2.420	0.530	0.712	0.030	0.653–0.771	<0.001^*∗*^

Sensitivity: true positive rate; specificity: true negative rate.

LR+, positive likelihood ratio; LR−, negative likelihood ratio.

LR + = sensitivity/(1 − specificity); LR − = (1 − sensitivity)/specificity.

Area: area under the curve; ^*∗*^*p* < 0.05 has significant statistical differences.

**Table 4:** The comparison of NIHSS and Barthel Index of ischemic stroke patients with retroflex and nonretroflex tongue.

Variable	Retroflex tongue		*p* value
	With (*N* = 185)	Without (*N* = 123)	
NIHSS ≥ 9, *N* (%)	81 (43.8)	93 (75.6)	<0.001^*∗*^
Barthel Index ≤ 17, *N* (%)	46 (24.9)	74 (60.2)	<0.001^*∗*^

Chi-square test or Fisher's exact test; ^*∗*^*p* < 0.05 has significant statistical differences.

## Abbreviations

RT: Retroflex tongue
TCM: Traditional Chinese medicine
NIHSS: NIH Stroke Scale.
